# Estimated prevalence of undiagnosed atrial fibrillation in the United States

**DOI:** 10.1371/journal.pone.0195088

**Published:** 2018-04-12

**Authors:** Mintu P. Turakhia, Jason Shafrin, Katalin Bognar, Jeffrey Trocio, Younos Abdulsattar, Daniel Wiederkehr, Dana P. Goldman

**Affiliations:** 1 Stanford University School of Medicine, Stanford, California, United States of America; 2 Precision Health Economics, Los Angeles, California, United States of America; 3 Pfizer Inc., New York, New York, United States of America; 4 Leonard D. Schaeffer Center for Health Policy & Economics, University of Southern California, Los Angeles, California, United States of America; University of Palermo, ITALY

## Abstract

**Introduction:**

As atrial fibrillation (AF) is often asymptomatic, it may remain undiagnosed until or even after development of complications, such as stroke. Consequently the observed prevalence of AF may underestimate total disease burden.

**Methods:**

To estimate the prevalence of undiagnosed AF in the United States, we performed a retrospective cohort modeling study in working age (18–64) and elderly (≥65) people using commercial and Medicare administrative claims databases. We identified patients in years 2004–2010 with incident AF following an ischemic stroke. Using a back-calculation methodology, we estimated the prevalence of undiagnosed AF as the ratio of the number of post-stroke AF patients and the CHADS_2_-specific stroke probability for each patient, adjusting for age and gender composition based on United States census data.

**Results:**

The estimated prevalence of AF (diagnosed and undiagnosed) was 3,873,900 (95%CI: 3,675,200–4,702,600) elderly and 1,457,100 (95%CI: 1,218,500–1,695,800) working age adults, representing 10.0% and 0.92% of the respective populations. Of these, 698,900 were undiagnosed: 535,400 (95%CI: 331,900–804,400) elderly and 163,500 (95%CI: 17,700–400,000) working age adults, representing 1.3% and 0.09% of the respective populations. Among all undiagnosed cases, 77% had a CHADS_2_ score ≥1, and 56% had CHADS_2_ score ≥2.

**Conclusions:**

Using a back-calculation approach, we estimate that the total AF prevalence in 2009 was 5.3 million of which 0.7 million (13.1% of AF cases) were undiagnosed. Over half of the modeled population with undiagnosed AF was at moderate to high risk of stroke.

## Introduction

Atrial fibrillation (AF) is a major cause of ischemic stroke, but often has minimal or no symptoms and therefore can be difficult to diagnose [1]. Current prevalence estimates are based on cohort studies and analyses of health care claims, but these approaches are unable to account for the prevalence of undiagnosed AF and therefore unable to measure true disease prevalence. Estimates of the prevalence of undiagnosed AF have been based on patient screening, and have varied widely from 1–2% of the general population [2–4] to over 15% among patients with a previous stroke [5].

In this paper, we propose an indirect back-calculation method to estimate the prevalence of undiagnosed AF. Back-calculation is a process whereby generally unobservable features of an event (i.e., undiagnosed AF leading to ischemic stroke) can be inferred. The methodology was first developed in the late 1980s for obtaining short-term projections of Acquired Immunodeficiency Syndrome [6]. For the present paper, we applied a non-parametric back-calculation methodology to estimate the prevalence of undiagnosed AF based on measuring the incidence of downstream complications (stroke) of the disease (AF), and then back-calculated total AF prevalence based on the attributable risk of the complication (stroke) to the disease (AF). We used retrospective health insurance claims data to derive AF prevalence estimates in both the working age adult and elderly (Medicare) populations.

## Methods

We performed a retrospective cohort study using 2004–2010 health insurance claims data from a commercial claims dataset representing a number of large, self-insured companies and administrative claims data from Medicare. The commercial claims data were used to study AF prevalence in the pre-Medicare, working adult population (aged 18–64 years); Medicare Limited Data Set was used to study the prevalence of AF in older adults (age ≥65 years). Data from commercial and Medicare administrative claims databases were fully anonymized before the authors accessed them. Our sample was restricted to U.S. residents aged ≥18 years with continuous enrollment for at least 12 month before and 15 months after the fourth quarter of 2009.

**Fig 1:**
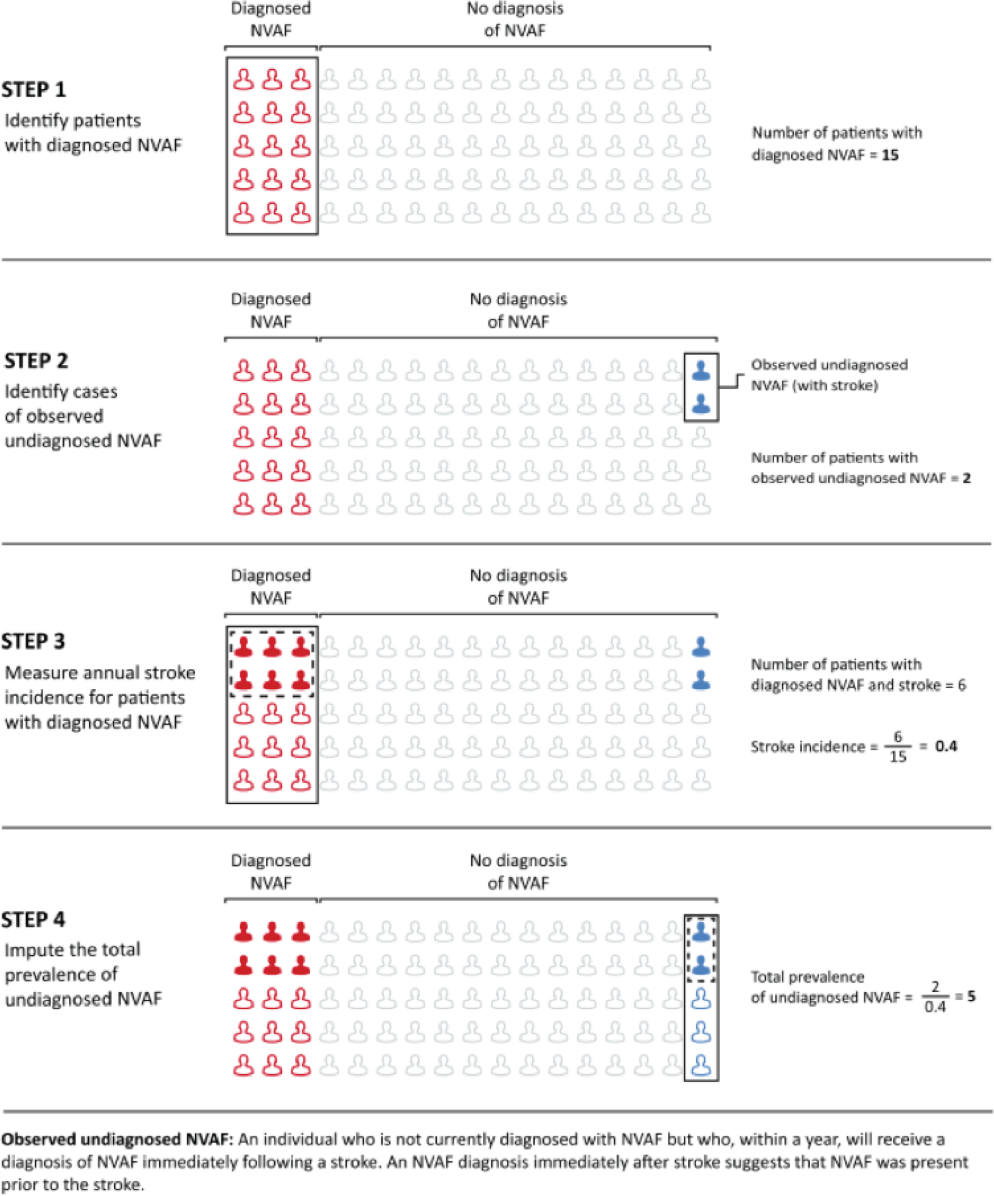
Imputing the prevalence of undiagnosed atrial fibrillation via back-calculation.

We estimated the prevalence of undiagnosed AF using a four-step back-calculation methodology (Fig 1) [7]. Step 1 identified patients with diagnosed AF at any point between 2004 and 2009. Patients had an AF diagnosis if they had an International Classification of Diseases, Ninth Revision (ICD-9-CM) code for atrial fibrillation (427.31) on ≥1 hospital inpatient, or on ≥2 hospital outpatient or physician visit claims during this time [8]. To minimize rule-out diagnoses, we did not count medical claims submitted by durable medical equipment providers, home health agencies, laboratories, or non-physician providers such as dentists or physical therapists [9, 10]. Furthermore as our back-calculation uses stroke event rates among patients with nonvalvular AF; we did not consider patients with valvular or transient AF. Patients with valvular AF were identified by having a diagnosis for a heart valve replacement or mitral valve stenosis (ICD-9-CM codes 42.2, 394.x, 396.1, 396.2, 396.8, or 746.5) or a procedure for a valve replacement (CPT codes 33405, 33420, 33422, 33425–33427, 33430, or 33496; ICD-9 procedure codes 35.0x, 35.1x, or 35.2x). Patients with transient AF were identified by an AF diagnosis appearing within 12 months after hyperthyroidism (ICD-9-CM codes 242.x) or within 30 days after coronary artery bypass surgery (ICD-9-CM codes 36.10 or 36.19), pericardial surgery (ICD-9-CM codes 37.10–37.12, 37.24, 37.25, 37.31–33, 37.35, or 37.40) or structural cardiac repair surgery (ICD-9-CM codes 35.31–35.39, 35.41–35.42, 35.50–35.54, 35.60–35.63, or 35.70–36.73) [11].

Step 2 identified patients who: (i) had no AF diagnosis between 2004–2009 while enrolled, (ii) had a stroke in 2010, and (iii) were subsequently diagnosed with non-valvular AF within 3 months after the stroke (“observable” cases of undiagnosed AF). Patients were classified as having an ischemic stroke if there was an inpatient or emergency room claim with a primary ICD-9-CM code of 433.01, 433.11, 433.21, 433.31, 433.81, 433.91; 434 or 436. This approach to identify patients with ischemic stroke has been previously validated with a sensitivity of 74%, specificity of 95%, and a positive predictive value of 88% [12]. As administrative coding of ischemic stroke has been shown to have limited sensitivity, we performed a sensitivity analysis in which we defined stroke to include ischemic stroke, hemorrhagic stroke (ICD-9-CM codes 430.x, 431.x, 432.0–432.9) and transient ischemic attack (TIA) (ICD-9-CM codes 435.x). Patients with post stroke non-valvular AF were identified as above using the appropriate timeframe. Because AF may cause stroke, but stroke is generally not considered to cause AF, we assumed that these individuals with AF coded soon after stroke are “observed” cases of undiagnosed AF. To account for the fact that some AF cases may appear spontaneously and not be the direct cause of a stroke, we adjusted the number of cases with stroke followed by AF by the rate of quarterly AF incidence among patients with no prior AF diagnosis.

Step 3 assigned stroke risk to patients with AF based on CHADS_2_ scores. We measured each patient’s CHADS_2_ score based on whether the patient had at least one claim for congestive heart failure (ICD-9 codes 428.x), hypertension (ICD-9-CM codes 401.x, 402.x, 403.x, 404.x, 405.x), diabetes (ICD-9-CM codes 250.x), or a previous stroke (ICD-9-CM codes 433.x1, 434.x1, 435.x). Furthermore, we used CHADS_2_ rather than CHA_2_DS_2_-VASc scores as there were very few people in the working-age population with low CHA_2_DS_2_-VASc scores who experienced AF after a stroke, leading to highly unstable estimates. Then we approximated stroke risks for patient with stroke and followed by an AF diagnosis using all-cause stroke risk by CHADS_2_ score among patients with diagnosed AF but not on anticoagulation from the literature [13]. As a sensitivity analysis, we used stroke incidence rates derived from our data (both commercial and Medicare) of patients with diagnosed AF, which include patients who are treated, untreated, and variably treated with anticoagulation.

Step 4 divided the number of observable cases (by CHADS_2_ score) of undiagnosed AF from Step 2 by the all-cause CHADS_2_-specific stroke probabilities determined in Step 3 to estimate the total number of undiagnosed AF cases.

To estimate the total prevalence of undiagnosed AF in the U.S. population, we adjusted our results according to the age/gender composition of the population [14]. As our measure of the prevalence of undiagnosed AF is a ratio of “observable” undiagnosed AF cases after stroke and the probability of stroke conditional on an AF diagnosis, confidence intervals were constructed using a bootstrapping method, a method well-suited for constructing confidence intervals for ratios [15].

We conducted three sensitivity analyses. First, we measured stroke incidence rates among AF patients from our claims data rather than the literature. Second, we replicated the analysis but defined a stroke more broadly to include ischemic stroke, transient ischemic attack, and hemorrhagic stroke. Third, we measured stroke incidence stratified by CHA_2_DS_2_-VASc score rather than CHADS_2_ score, based on stroke risk as measured in previous studies [16].

## Results

**Fig 2:**
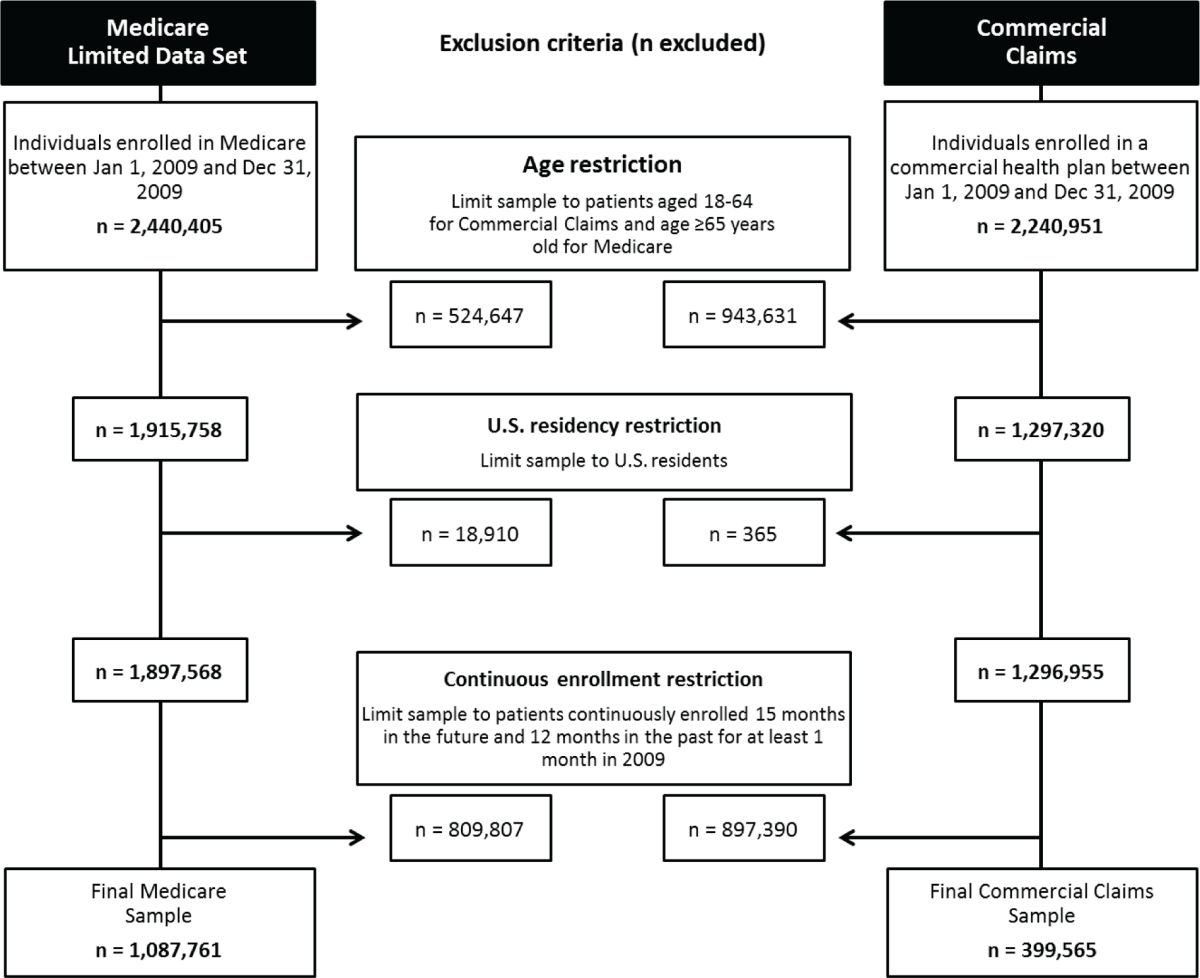
Cohort diagram.

Of the initial sample of 2.4 million Medicare beneficiaries and 2.2 million commercially insured individuals in our database as of 2009, we identified 1.1 million Medicare and 400,000 privately insured beneficiaries who met the inclusion criteria (Fig 2).

Patients with observed (diagnosed) AF were older, more likely to be male, and had higher CHADS_2_ scores compared to patients without AF (Table 1). Among elderly patients who had a stroke, patients diagnosed with AF prior to the stroke were 1.8 years older (p<0.001) with a CHADS_2_ score that was 0.7 points (p<0.001) higher than patients who were first diagnosed with AF within 3 months after the stroke (Table 2).

**Table 1:** Baseline characteristics of patients with observed and unobserved AF.

	Working Age Adults (Age 18–64 years)			Elderly (Age ≥ 65 years)		
	AF Observed	AF Not Observed	p-value	AF Observed	AF Not Observed	p-value
*Mean Age*, *y*	56.1 (55.9‒56.3)	47.9 (47.9‒48.0)	<0.001	79.2 (79.1‒79.2)	75.9 (75.9‒75.9)	<0.001
*Female*, *%*	32.7 (31.1‒34.3)	56.3 (56.1‒56.4)	<0.001	53.1 (52.8‒53.4)	61.0 (60.9‒61.1)	<0.001
*Comorbidities*, *%*						
Congestive Heart Failure	7.4 (6.5‒8.3)	0.3 (0.3‒0.3)	<0.001	23.3 (23.0‒23.5)	5.0 (4.9‒5.0)	<0.001
Diabetes	19.5 (18.2‒20.9)	7.5 (7.4‒7.6)	<0.001	27.6 (27.3‒27.8)	22.9 (22.8‒23.0)	<0.001
Hypertension	36.3 (34.7‒37.9)	15.8 (15.6‒15.9)	<0.001	64.0 (63.7‒64.3)	53.3 (53.2‒53.4)	<0.001
Previous Stroke/TIA	1.3 (0.9‒1.7)	0.2 (0.2‒0.3)	<0.001	4.7 (4.6‒4.9)	2.1 (2.1‒2.1)	<0.001
Vascular Disease	4.7 (4.0‒5.4)	0.8 (0.8‒0.9)	<0.001	17.3 (17.0‒17.5)	9.8 (9.8‒9.9)	<0.001
Stroke Risk, y						
CHADS_2_ Score	1.2 (1.1‒1.2)	0.4 (0.4‒0.4)	<0.001	2.6 (2.6‒2.6)	1.8 (1.8‒1.8)	<0.001
CHA_2_DS_2_-VASc Score	1.6 (1.6‒1.7)	1.0 (1.0‒1.0)	<0.001	4.6 (4.6‒4.6)	3.6 (3.6‒3.6)	<0.001
*Sample Size*, *y*						
Number of Unique Patient Quarters	8,391	1,004,196		242,386	2,509,475	
Number of Unique Individuals	2,820	396,745		84,933	1,002,828	

**Table 2:** Baseline characteristics of patients with AF diagnosed before stroke versus after stroke.

	Working Age Adults (Age 18–64 years)			Elderly (Age ≥ 65 years)			
	AF diagnosed before stroke	AF diagnosed after stroke	p-value	All	AF diagnosed before stroke	AF diagnosed after stroke	p-value
*Mean Age*, *y*	56.8 (54.5–59.2)	55.9 (53.8–58.0)	0.455	76.2 (76.2–76.2)	80.9 (80.5–81.3)	79.1 (78.6–79.7)	<0.001
*Female*, *%*	46.7 (29.0–64.5)	42.9 (25.4–60.3)	0.803	60.3 (60.2–60.4)	60.0 (57.1–62.9)	61.9 (58.1–0.7)	0.560
*Comorbidities*, *%*							
Congestive Heart Failure	14.1 (1.7–26.5)	7.7 (-1.7–17.1)	0.566	6.6 (6.5–6.6)	29.2 (26.6–31.9)	11.6 (9.1–0.1)	<0.001
Diabetes	28.3 (12.2–44.3)	27.5 (11.7–43.2)	0.957	23.3 (23.3–23.4)	32.9 (30.1–35.6)	29.8 (26.2–0.3)	0.309
Hypertension	39.1 (21.8–56.5)	37.4 (20.3–54.4)	0.908	54.2 (54.1–54.3)	71.1 (68.5–73.8)	64.6 (60.9–0.7)	0.034
Previous Stroke/TIA	5.4 (-2.6–13.5)	7.7 (-1.7–17.1)	0.824	2.3 (2.3–2.4)	13.0 (11.0–15.0)	12.3 (9.8–0.1)	0.795
Vascular Disease	5.4 (-2.6–13.5)	5.5 (-2.6–13.5)	0.995	10.5 (10.4–10.5)	19.7 (17.3–22.0)	15.3 (12.5–0.2)	0.100
Stroke Risk, y							
CHADS_2_ Score	1.6 (1.2–2.0)	1.4 (0.9–1.9)	0.611	1.86 (1.86–1.87)	3.2 (3.1–3.3)	2.5 (2.4–2.6)	<0.001
CHA_2_DS_2_-VASc Score	2.3 (1.9–2.7)	2.0 (1.4–2.6)	0.391	3.72 (3.72–3.73)	5.3 (5.2–5.4)	4.5 (4.4–4.6)	<0.001
*Sample Size*, *y*							
Number of Unique Patient Quarters	92	91		2,751,861	3,019	1,670	
Number of Unique Individuals	31	31		1,087,761	1,102	629	

For working adults aged 18–64, the prevalence of diagnosed AF in our data was 0.83% (95% CI: 0.79%-0.86%), whereas 8.70% (95% CI: 8.62%-8.78%) of elderly adults had diagnosed AF (Table 3). The latter figure was comparable to a 2007 estimate of AF prevalence among Medicare beneficiaries (8.58%) calculated in Piccini et al [17]. Extrapolating the general U.S. population, we estimated that 4.63 million individuals had diagnosed AF, of which 1,293,600 (95% CI: 1,238,300–1,347,100) were working age adults and 3,338,500 (95% CI: 3,305,000–3,372,200) were elderly.

**Table 3:** Prevalence of diagnosed and undiagnosed AF.

		Working Age Adults (Age 18–64 years)			Elderly (Age ≥ 65 years)		
		Diagnosed	Undiagnosed	AF % undiagnosed	Diagnosed	Undiagnosed	AF % undiagnosed
*All*	N	1,293.6 (1,238.3, 1,347.1)	163.5 (17.7, 400.0)	11.20%	3,338.5 (3,305.0, 3,372.2)	535.4 (331.9, 804.4)	13.80%
	%	0.83 (0.79, 0.86)	0.09 (0.01, 0.22)		8.70 (8.62, 8.78)	1.32 (0.86, 1.94)	
*Gender*							
Male	N	907.8 (873.3, 940.6)	99.8 (7.1, 254.4)	12.00%	1,741.4 (1,724.1, 1,759.2)	237.4 (138.2, 373.6)	9.90%
	%	1.27 (1.22, 1.31)	0.10 (0.01, 0.25)		10.28 (10.18, 10.38)	1.30 (0.80, 2.00)	
Female	N	385.8 (365.1, 406.4)	63.7 (10.6, 145.6)	15.70%	1,597.1 (1,580.9, 1,613.1)	298.0 (193.7, 430.8)	14.20%
	%	0.48 (0.46, 0.51)	0.08 (0.01, 0.19)		7.66 (7.59, 7.73)	1.33 (0.90, 1.89)	
*Age*							
18–54	N	624.6 (596.8, 650.4)	106.5 (6.4, 275.3)	14.60%			
	%	0.38 (0.36, 0.39)	0.06 (0.00, 0.16)				
55–59	N	283.9 (271.9, 296.0)	24.1 (2.9, 58.4)	7.80%			
	%	1.35 (1.29, 1.41)	0.11 (0.01, 0.28)				
60–64	N	385.1 (369.7, 400.6)	32.8 (8.4, 66.4)	7.90%			
	%	2.21 (2.12, 2.30)	0.19 (0.05, 0.37)				
65–69	N				563.3 (555.5, 571.2)	151.6 (68.2, 257.9)	21.20%
	%				4.04 (3.99, 4.10)	1.11 (0.50, 1.88)	
70–74	N				621.6 (615.2, 628.4)	128.8 (76.9, 203.0)	17.20%
	%				6.26 (6.20, 6.33)	1.32 (0.79, 2.07)	
75–79	N				699.5 (692.6, 705.6)	61.2 (44.8, 82.0)	8.00%
	%				9.48 (9.39, 9.57)	0.83 (0.61, 1.12)	
80–84	N				726.7 (720.1, 733.5)	92.7 (68.2, 123.8)	11.30%
	%				12.37 (12.26, 12.48)	1.62 (1.19, 2.15)	
≥85	N				727.4 (721.4, 733.4)	101.2 (73.7, 137.7)	12.20%
	%				14.28 (14.17, 14.40)	2.00 (1.46, 2.71)	
*Sensitivity #1*	N	1,293.6 (1,238.3, 1,347.1)	847.6 (46.4, 2,106.1)	39.60%	3,338.5 (3,305.0, 3,372.2)	2,003.0 (1,579.1, 2,456.1)	37.50%
(Stroke Risk)*	%	0.83 (0.79, 0.86)	0.47 (0.04, 1.15)		8.70 (8.62, 8.78)	5.06 (4.08, 6.10)	
*Sensitivity #2*	N	1,293.6 (1,238.3, 1,347.1)	360.7 (36.1, 865.7)	21.80%	3,338.5 (3,305.0, 3,372.2)	1140.1 (1009.5, 1314.3)	25.46%
*(Stroke Definition)*^†^	%	0.83 (0.79, 0.86)	0.20 (0.02, 0.48)		8.70 (8.62, 8.78)	2.88 (2.55–3.32)	

*: Sensitivity analysis #1 uses stroke probabilities from diagnosed AF in the claims data to conduct the back-calculation.

^*†*^: Sensitivity analysis #2 defines stroke as ischemic stroke, hemorrhagic stroke, or TIA, whereas as the baseline approach only uses ischemic stroke.

Note: The "%" results are estimated prevalence rates as measured in the sample data. The "N" results are the estimated U.S. prevalence after extrapolating the results to the U.S. population and adjusting for differences between the age/gender composition of the sample and the broader U.S. population. All "N" results are expressed in 1,000's.

Applying the back-calculation methodology, we estimated that 163,500 (95% CI: 17,700–400,000) working age adults had undiagnosed AF. Although the prevalence of undiagnosed AF among working age adults was rare overall (0.09% prevalence; 95% CI: 0.01%-0.22%), 11.2% of all persons with AF in the working age population were undiagnosed. We estimated that 1.32% (95% CI, 0.86%-1.94%) or 535,400 (95% CI: 331,900–804,400) elderly patients had undiagnosed AF. Among the elderly, 13.8% of persons with AF were undiagnosed.

**Fig 3:**
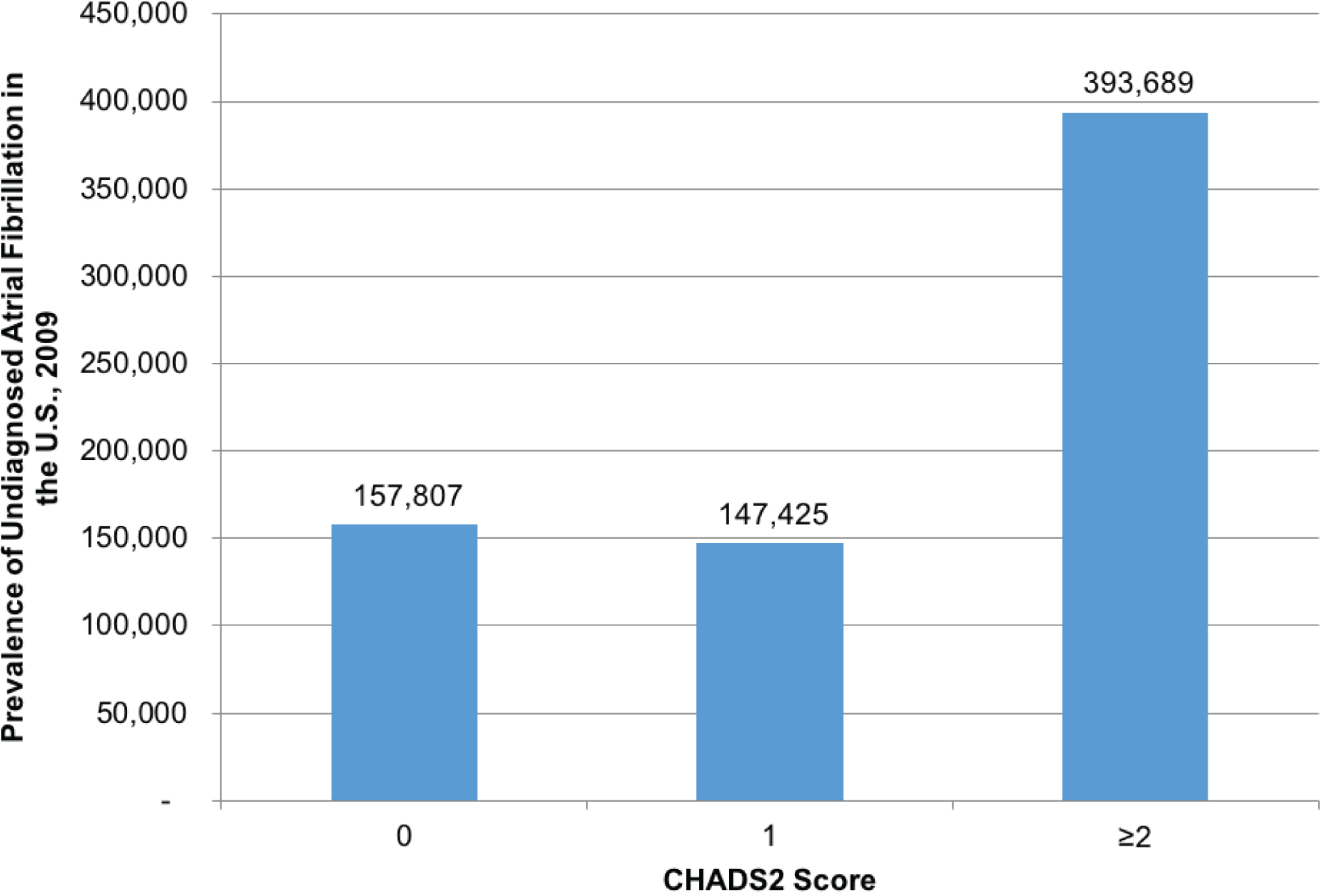
Distribution of CHADS_2_ scores among patients with undiagnosed atrial fibrillation.

The total prevalence (both diagnosed and undiagnosed) of AF in the U.S. was estimated to be 5.33 million (2.4% of adults) comprised of 1,457,100 (95% CI: 1,218,500–1,695,800) working age adults and 3,873,900 (95% CI: 3,675,200–4,702,600) patients aged ≥65 years representing 0.92% and 10.0% of these populations, respectively. Of the total 5.33 million patients with AF, 698,900 (13.1%) were undiagnosed. Although males were more likely to have AF, a larger proportion of female patients with AF were undiagnosed. Similar to patients with diagnosed AF, undiagnosed AF increased with age. Overall, 56% of undiagnosed AF cases had a CHADS_2_ score ≥2, and 77% had CHADS_2_ ≥1 (Fig 3).

When we applied CHADS_2_-specific stroke incidence rates derived from our datasets of patients with diagnosed AF, our estimated prevalence of undiagnosed AF increased. Under this alternative assumption, the estimated prevalence of undiagnosed AF was 0.85 million (0.47%; 95% CI: 0.04%-1.15%) among working age individuals and 2.0 million (5.06%; 95% CI: 4.08%-6.10%) among the elderly, a total of 2.85 million individuals. When we defined stroke in the claims data using ischemic stroke, hemorrhagic stroke, or TIA, the prevalence of undiagnosed AF was 0.20% (95% CI: 0.02%-0.48%) for working age adults the 2.88% (95% CI: 2.55%-3.32%) for elderly adults. In our third sensitivity analysis using CHA_2_DS_2_-VASc scores to predict stroke risk rather than CHADS_2_ scores, we found that the results for our commercial population were unstable due to the small sample size of patients with stroke and the increased number of potential scores (i.e., 0 to 9 for CHA_2_DS_2_-VASc rather than 0 to 6 for CHADS_2_). Among the elderly individuals, however, we estimated that 1.22 million (3.08%; 95% CI: 2.11% to 4.15%) of people aged 65 years and older had undiagnosed atrial fibrillation.

## Discussion

The estimated prevalence of undiagnosed AF in the U.S. in 2009 was 700,000 while the total prevalence of AF was 5.3 million. One in eight patients with AF was undiagnosed. Furthermore, over half of the population with undiagnosed AF was at moderate to high risk of stroke.

Our total prevalence estimate of 5.3 million (2.4% of all adults) for 2009 is markedly higher than prior projections of 2.2–2.7 million (1.0–1.2%) in 2009–2010 from the American Heart Association (AHA) Statistical Update [18] and estimates of 3.0 million (1.6%) by Naccarelli and colleagues [19]. The AHA projections are derived from cross-sectional studies of 1996–1997 Kaiser Permanente Northern California enrollees [11] while Naccarelli and colleagues used a claims-based method, neither accounting for undiagnosed AF. However, our baseline projections are aligned with a model-based projection (2.5%) derived from a 1990–2000 community sample of Olmsted County, Minnesota residents [20].

Prior estimates of undiagnosed AF prevalence come primarily from AF screening studies, reporting 1.7% in a general European population [3] and 2.3–15.1% among patients with diabetes, hypertension, or previous stroke or TIA [5, 21].

These data show appreciable rates of undiagnosed AF such that targeted screening strategies could prove beneficial, particularly in subgroups of older (age ≥65) patients or in patients with multiple CHADS_2_ risk factors, in whom prevalence is substantially higher. Our results indicated that the prevalence of undiagnosed AF increases from 0.11% of the population of patients with a CHADS_2_ score of zero to 0.73% of the population of patients with a CHADS_2_ score of 2 and up to 0.95%-1.39% of the population of patients with a CHADS_2_ score between 3 and 6. At the same time, our data indicated that routine full population screening may be of low yield, as the younger working age population had an undiagnosed AF prevalence of 0.09%.

There are several limitations to our analysis. First, our back-calculation methodology assumed that all AF detected in the three months after ischemic stroke was present prior to stroke. We believe this to be reasonable and biologically plausible, and our estimates showed good calibration to known AF prevalence estimates. To address the case where AF appears spontaneously after stroke, we did adjust our prevalence estimates based on the baseline AF incidence in our data. Second, our model may have underestimated the prevalence of undiagnosed AF since a number of people with AF have silent strokes that would not be captured by claims data, have short episodes of AF that may not meaningfully increase stroke risk, or may have cryptogenic strokes that were in fact due to undiagnosed AF. Third, estimates were modeled from data sources that may not be truly representative of the U.S. population (e.g. Medicaid, uninsured/unemployed, Medicare Advantage patients, broader employee base are not well represented). Fourth, our model did not account for differences in stroke event rates by race or ethnicity and we assumed that stroke incidence rates among patients with undiagnosed AF were similar to those of patients with diagnosed AF. Fifth, data and funding available to the authors at the time of research was only through 2010. Finally, the estimates presented rely on the accuracy of the back-calculation model; population AF screening studies will be required to better estimate point prevalence of undiagnosed AF.

## Conclusion

Based on a model of back-calculating disease prevalence from ischemic stroke events, we estimate that the total AF prevalence in 2009 was approximately 5.3 million persons, which is substantially higher than prior estimates based on cohort and population studies. Of these patients with AF, about 700,000 persons were undiagnosed—about one in eight patients—corresponding to 0.31% of the total US adult population.
